# Immune Modulatory Short Noncoding RNAs Targeting the Glioblastoma Microenvironment

**DOI:** 10.3389/fonc.2021.682129

**Published:** 2021-08-31

**Authors:** Jun Wei, Eli Gilboa, George A. Calin, Amy B. Heimberger

**Affiliations:** ^1^Department of Immunology, The University of Texas MD Anderson Cancer Center, Houston, TX, United States; ^2^Department of Microbiology & Immunology, Dodson Interdisciplinary Immunotherapy Institute, Sylvester Comprehensive Cancer Center, University of Miami, Miami, FL, United States; ^3^Departments of Translational Molecular Pathology, The University of Texas MD Anderson Cancer Center, Houston, TX, United States; ^4^Department of Neurological Surgery, Feinberg School of Medicine, Northwestern University, Chicago, IL, United States

**Keywords:** noncoding RNAs, tumor microenvironment, microRNA, siRNA, aptamer, antisense oligonucleotide, glioblastoma

## Abstract

Glioblastomas are heterogeneous and have a poor prognosis. Glioblastoma cells interact with their neighbors to form a tumor-permissive and immunosuppressive microenvironment. Short noncoding RNAs are relevant mediators of the dynamic crosstalk among cancer, stromal, and immune cells in establishing the glioblastoma microenvironment. In addition to the ease of combinatorial strategies that are capable of multimodal modulation for both reversing immune suppression and enhancing antitumor immunity, their small size provides an opportunity to overcome the limitations of blood-brain-barrier (BBB) permeability. To enhance glioblastoma delivery, these RNAs have been conjugated with various molecules or packed within delivery vehicles for enhanced tissue-specific delivery and increased payload. Here, we focus on the role of RNA therapeutics by appraising which types of nucleotides are most effective in immune modulation, lead therapeutic candidates, and clarify how to optimize delivery of the therapeutic RNAs and their conjugates specifically to the glioblastoma microenvironment.

## Introduction

Glioblastoma cells would not survive without close connection and dependence on their adjacent molecular and cellular components. Multilevel and complicated communication between glioblastoma cells and nonmalignant cells promotes a permissive microenvironment for gliomagenesis. The glioblastoma microenvironment can be taken as a local niche comprising glioma cells, immune cells, parenchymal cells, and their associated molecular factors and subcellular vesicles. Microglia, a major type of parenchymal cells, contribute significantly to the brain tumor mass and immunosuppressive microenvironment. In addition, these cells can secrete a number of factors together with glioblastoma cells and nonneoplastic astrocytes that have an effect on glioblastoma progression.

Heterogenous molecular factors contribute to the complexity of the glioblastoma microenvironment and emphasize the importance of local niche influence to the tumor cells. An important contributor to these molecular factors is short noncoding RNAs (ncRNAs) including small noncoding microRNAs (miRNAs) and small interfering RNAs (siRNAs) carried by extracellular vehicles (EVs). miRNAs account for a large portion of the human transcriptome in the glioblastoma cells and their surrounding cells, and these miRNAs regulate numerous hallmarks of glioblastoma such as proliferation, invasion, immune escape and resistance to treatment (1). The development of various RNA agents including siRNA, antisense oligonucleotides (ASO), and aptamers for specific gene targeting and knockdown offers enormous therapeutic potential (2). In this manuscript, we discuss the current state of knowledge of miRNA immune regulatory functions and the impact of immunomodulatory miRNAs on the glioblastoma microenvironment. Next, we will review possible use of miRNAs, their analogue siRNAs, and aptamers as antiglioblastoma therapeutics. Finally, we will discuss potential RNA nucleotide therapeutics targeting immunomodulatory pathways and survey effective strategies for delivery to the glioblastoma microenvironment.

## miRNAs in the Glioblastoma Microenvironment

### miRNA Roles in Glioblastoma Pathobiology

Our understanding of gene expression modulation evolved upon the discovery of the role of miRNA as an epigenetic regulator. miRNAs at 21–23 nucleotide long can suppress target gene expression by binding to the 3′ untranslated regions (UTRs) of mRNA with partial complementarity base-pairing (3). At present, around 2,500 human miRNAs have been identified that act in this capacity (4). miRNA and mRNA interactions can be complex: a mRNA can be modulated by multiple miRNAs and a miRNA can target many different mRNAs. Thus, it is not surprising that miRNAs play important roles in glioblastoma initiation and progression. miRNAs have been indicated as critical regulators of glioblastoma stem cell maintenance (5), epigenetic regulation (6), tumorigenesis (7), oncogenic pathways, and migration (8, 9). Furthermore, miRNA is involved in regulating radio- and chemotherapy resistance and sensitivity and may serve as biomarkers for diagnosis and outcome (10, 11).

### Glioblastoma Cell-Associated miRNAs

Upregulated miRNAs in glioblastoma cells can act as oncogenes (oncomiRs) and silence onco-suppressor genes. A prototypical example and one of the first oncomiRs identified is miR-21 which is involved in malignant processes by targeting genes important for proliferation, cell survival, invasion, and treatment resistance (12). Other upregulated miRNAs such as the miR-17-92 cluster, miR-10b, and miR-15b have been investigated in preclinical studies and shown to be indispensable for tumor initiation. As such, oncomiR blockade in glioma cells could activate numerous tumor suppressor genes (13) and also restore immune surveillance of the glioblastoma (14, 15). Liu et al., for example, demonstrated that miR-340-5p suppression in glioblastoma cell enhanced M2 macrophage polarization and macrophage recruitment to the glioblastoma microenvironment (16), suggesting restoration of miR-340-5p could be a potential strategy to reverse immune suppression mediated by M2 macrophages.

In contrast, some miRNAs may function as tumor suppressors and could be therapeutically reconstituted. For instance, the miR-1, miR-7, miR-34a, miR-124, miR-128, miR-138, and miR-181 family are a group of suppressor miRNAs inhibiting glioblastoma progression when they are overexpressed (8, 17, 18). These miRNAs not only directly target oncogenic/tumor suppressor pathways in glioma cells but also exert broad regulatory effects on the immune system. Our group found that miR-124 inhibited multiple targets in the signal transducer and activator of transcription 3 (STAT3) signaling pathway and reversed immune dysfunction of T cells induced by glioblastoma stem cells (GSCs) (19). We also showed that miR-138 can target multiple immune checkpoint molecules such as CTLA-4 and programmed cell death protein 1 (PD-1) to inhibit tumor-infiltrating Treg*s. In vivo* administration of miR-138 suppressed tumor development and significantly prolonged survival time of immune-competent glioma-bearing mice, but not immune-deficient mice (20), indicating the pivotal role of miR-138 in immunological tumor surveillance.

One of the key mechanisms for intercellular communication in the tumor microenvironment are exosomes that contain a wide variety of miRNAs (12–16, 21). Exosomal miR-21 has been shown to be an important mediator of immune cell reprogramming by glioblastoma cells to create a niche favorable for cancer progression (14). miR-1246 has been identified as the most enriched miRNA in glioblastoma-derived exosomes and mediates glioblastoma-induced protumorigenic macrophage formation by targeting TERF2IP and subsequently activating the STAT3 pathway (22). miR-214-5p, another glioblastoma-derived exosomal miRNA, mediates proinflammatory responses by targeting CXCR5 in primary microglia upon lipopolysaccharide stimulation (23). Furthermore, exosomal miR-29a and miR-92a from glioblastoma cells promotes the proliferation and immunosuppressive phenotype of glioblastoma-infiltrating macrophages (GIMs) by targeting protein kinase cAMP-dependent type I regulatory subunit alpha and high-mobility group box transcription factor 1 (24). Given this is an emerging area of investigation, it is likely that almost every key pathway and mechanism elucidated for gliomagenesis will have a network of miRNA control.

### Glioblastoma-Infiltrating Macrophages Associated miRNAs

Secondary to an immunosuppressive microenvironment, glioblastoma patients are deficient in antitumor immunity leading to malignant progression and resistance to treatment. GIMs, the most frequent infiltrating immune cell subset (25), originate from peripheral blood monocytes in response to tumor-derived chemokines. Upon entry into the glioblastoma, the macrophages adopt a M0/M2 phenotype with the capacity to promote tumor cell invasion and exert immune suppression through factors like tumor growth factor-β (TGFβ) (26, 27). We have shown that miR-142-3p, by modulating the TGFβ signaling, inhibits the M2 phenotype of GIMs, and systemic *in vivo* administration of miR-142-3p induces antiglioma immune function (28). Ishii et al. reported that exogenous expression of miR-130a and miR-145 in myeloid-derived suppressor cells (MDSCs) decreased tumor metastasis through downregulation of TGFβ receptor II (TβRII) and related immune suppressive cytokines (29). Reciprocally, GIM-derived exosomal miR-22-3p, miR-27a-3p, and miR-221-3p can promote the proneural-to-mesenchymal transition of GSCs by simultaneously targeting CHD7 (30). miR-21 is also enriched in GIM-derived exosomes and mediates temozolomide (TMZ) resistance for which the STAT3 inhibitor pacritinib could overcome this resistance by downregulating miR-21 (31). Moreover, the downregulation of miR-21 promotes M1 macrophage polarization (32). As such, miR-21 deregulation is an operational mechanism in both GIMs and glioblastoma cells that modulates multiple oncogenic molecules, and signaling pathway such as STAT3 and anti-miR-21 strategies could be therapeutically developed.

### Astrocyte-Associated miRNAs

In addition to the crosstalk between glioblastoma and immune cells, astrocytes also contribute to tumor growth, invasion, and immune suppression (33). For example, miR-19a is transferred from astrocytes to metastatic cancer cells in the central nervous system (CNS). Zhang et al. have demonstrated that exosomal transfer of miR-19a downregulates PTEN and subsequently promotes cytokine chemokine ligand 2 (CCL2) secretion. CCL2 recruits macrophages that contribute to immune resistance (34). Another astrocyte-associated miRNA, miR-10b, is overexpressed in gliomas and brain metastasis, and this miRNA can induce astrocyte transformation (13). Targeting astrocytes and their associated miRNAs is an emerging strategy for potentially treating CNS tumors.

### Oligodendrocyte-Associated miRNAs

Oligodendrocytes have important protecting roles because they produce myelin for neuron protection. Being a major cell population in the glioblastoma microenvironment, oligodendrocytes interact with astrocytes and microglia and participate in the formation of a tumor permissive niche (35, 36). Oligodendrocyte progenitor cells (OPCs) are the largest proliferating population in the CNS, and together with GIMs, are enriched at the infiltrating edge (37). Several miRNAs such as miR-219, miR-129-2, and miR-338 have higher expression at the infiltrating edge and are involved in oligodendrocyte differentiation (38). Negative regulators of oligodendrocyte differentiation such as SOX6, HES5, PDGFRA, and ZFP238 are suppressed by these miRNAs (39). One of the important functions of miR-219 is to mediate OPC differentiation to oligodendrocytes (40). Additionally, miR-219 indirectly promotes receptor tyrosine kinase signaling activity by targeting and inhibiting epidermal growth factor receptor expression. Thus, the evidence points to the involvement of oligodendrocyte-associated miRNAs in the glioblastoma microenvironment and warrants further study to determine their exact targets mediating the crosstalk of the OPCs and tumor cells.

### Endothelial Cell-Associated miRNAs

**Figure 1:**
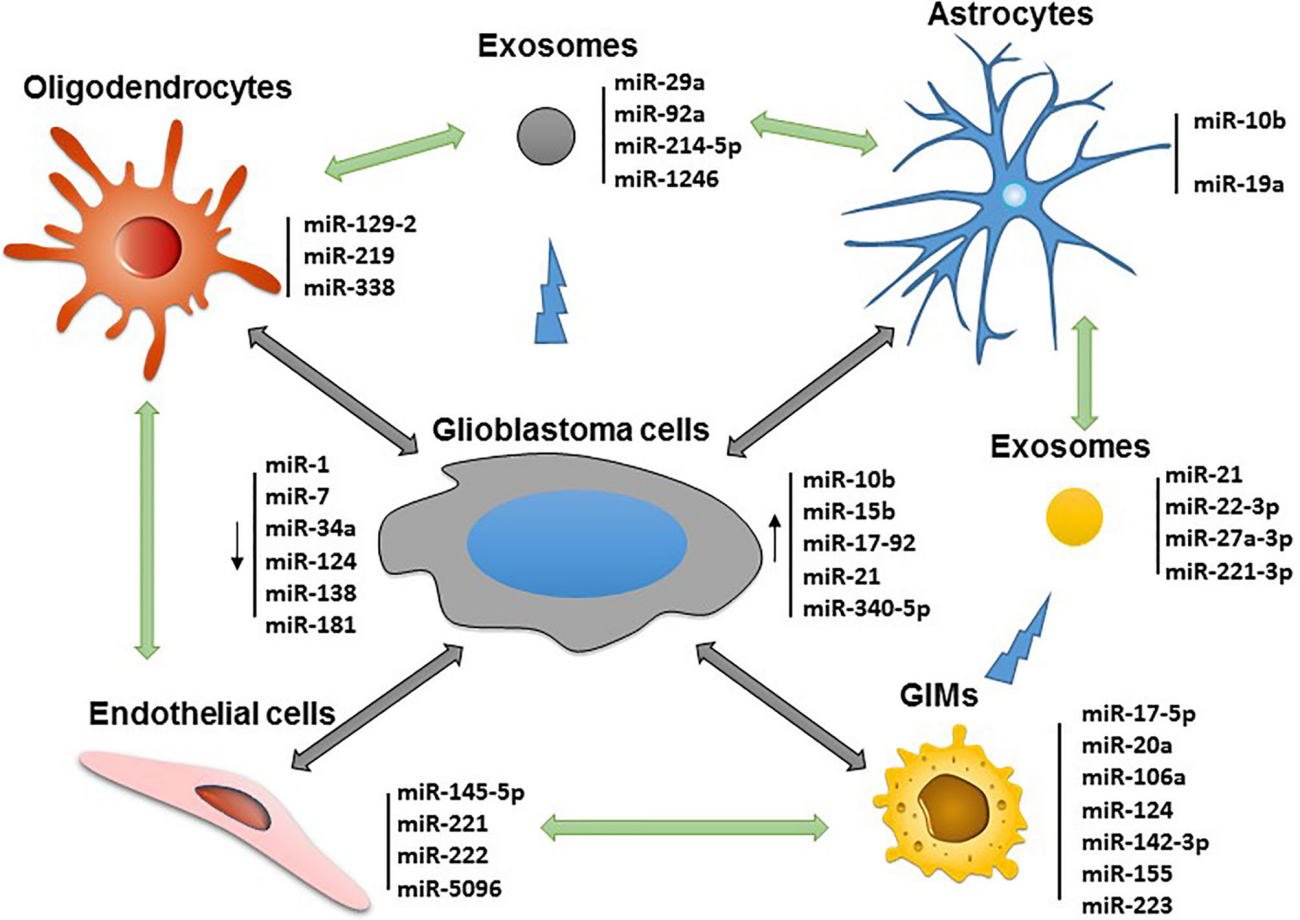
Potential miRNAs that can be targeted for immune modulation and therapeutic application in the glioblastoma microenvironment.

Glioblastoma is highly vascularized, and its invasion and outgrowth rely on a nutrient supply by acquiring new blood vessel formation (41). Endothelial cell proliferation from the tumor is a direct measure of its malignancy. GSC-associated exosomes are capable of inducing angiogenesis of endothelia cells mediated by miR-21 (42). Some endothelial cell-associated miRNAs such as miR-145-5p and miR-5096 can transfer between human microvascular endothelial cells (HMECs) and glioblastoma cells through gap junctions. In the process, miR-145-5p is downregulated in early gliomagenesis and acts as a tumor suppressor when passing from HMECs into glioma cells, whereas miR-5096 is transferred from glioma cells into HMECs and promotes angiogenesis (43). Finally, the miR-221/222 cluster has also been shown to enhance angiogenesis and silencing attenuates angiogenesis by inhibiting the JAK/STAT pathway (44). All these targetable miRNAs associated with the various cell lineages in the glioblastoma microenvironment are depicted in **Figure 1**.

## Small Interfering RNAs and Antisense Oligonucleotides

In addition to the use of miRNAs, siRNA, which are 21–27 base pair double-stranded oligonucleotides, are another treatment modality for inhibiting protein synthesis at the posttranscriptional level. Although both miRNA and siRNA strategies are capable of BBB penetration, miRNA has the therapeutic advantage of target networks which would be beneficial in complex heterogenous cancers such as glioblastoma. However, miRNA off target effects remains a substantial concern. In contrast, siRNA approaches have much greater specificity that is counterbalanced by tumor plasticity and escape mechanisms.

### Potential Candidates of Immune Modulatory siRNAs Targeting the Glioblastoma Microenvironment

#### Vascular Endothelial Growth Factor

Abnormal vasculature is enriched in glioblastoma as a consequence of upregulated angiogenic factors such as VEGF. Increased VEGF causes new blood vessels to form within the tumor *via* angiogenesis and the associated proliferation of endothelial cells (45). The resulting vascular networks display increased vessel permeability and enlarged vessel size that results in plasma leakage into the tumor tissue and disruption to the BBB. Together, these abnormalities induce inflammation, cerebral edema, and increased interstitial pressure. Thus, antiangiogenic VEGF treatments have been extensively investigated including monoclonal antibodies such as bevacizumab and small molecules targeting its receptor VEGFR (46). These agents have limitations based on short half-life, limited efficacy in patient overall survival improvement, and systemic toxicity. Some siRNA formulations targeting VEGF have been evaluated in early stage clinical trials, but this have not been advanced further (47, 48).

#### Programmed Cell Death Protein 1

T cells are present in the glioblastoma microenvironment, although at lower frequencies than GIMs (25). They have a profoundly exhausted phenotype characterized by expression of multiple immune checkpoint ligands (49, 50) likely accounting for their inability to control tumor growth. The lack of effective T-cell response is also highlighted by the ineffectiveness of checkpoint blockade immunotherapy in glioblastoma. Nonetheless, anti-PD-1 therapy achieves potent antiglioma activity in mouse glioma activity possibly through the depletion of PD-1+ macrophages and proinflammatory polarization in the glioblastoma microenvironment (51). A RNAi specific to the PD-1/PD-L1 pathway was delivered by a hemagglutinating virus of Japan-envelope—a nonreplicating viral vector that was capable of inhibiting immune suppression and eliciting antiglioma immune responses (52). Similarly, we showed that miR-138 could downregulate both CTLA-4 and PD-1 to inhibit tumor-infiltrating regulatory T cells (Tregs) and *in vivo* administration induced tumor reduction and prolonged the survival of immune syngeneic glioma-bearing mice (20).

#### Neuroligin 3

Neuronal activity is involved in glioblastoma growth and progression (53). In the normal brain microenvironment, neurons are strong mitogenic signalers stimulating the growth of neural and oligodendrocyte precursor cells—an important consideration in the role of stem/progenitor cells in glioblastoma (54). Elegant studies of neuronal activity conducted by Venkatesh et al. in xenograft glioma mouse models show that presynaptic and postsynaptic function is disrupted in the presence of glioma with microenvironmental neurolignin 3 (NLGN3) being hijacked to induce signaling through the PI3K/PTEN/AKT/mTOR pathway (55). Neurons and OPCs produce NLGN3 by cleavage of ADAM10 sheddase, so the inhibition of this enzyme blocks NLGN3 secretion into the tumor microenvironment and suppresses glioma outgrowth in preclinical models. Therefore, siRNAs targeting NLGN3/ADAM10 are promising for treating glioblastoma by modulating the interaction between neuronal cells and tumor cells (56).

#### Growth Differentiation Factor

Growth differentiation factor (GDF15) is highly expressed in glioblastoma as a secreted cytokine participate in regulating tumor cell proliferation and immunosuppression (57). GDF15 promotes GSC stemness by activating the leukemia inhibitor factor–STAT3 pathway (58). Thus, it represents a potential therapeutic target in glioblastoma treatment by siRNA targeting GDF15 or its cognate receptor GFRAL (the GDNF family receptor alpha like) (59).

#### O^6^-Methylguanine-DNA Methyltransferase

Temozolomide (TMZ) is the standard-of-care for glioblastoma and the other brain tumors, but many patients show limited response due to unmethylated O^6^-methylguanine-DNA methyltransferase **(**MGMT). Efforts to inhibit MGMT activity by systemic delivery of a siRNA have been made to silence the TMZ resistance gene MGMT. Wang et al. developed a MGMT siRNA nanoparticle that when combined with TMZ was found to reduce tumor growth and significantly extending survival in a GSC xenograft model relative to TMZ monotherapy (60). Like MGMT, other DNA damage response mediators such as ataxia-telangiectasia-mutated, ataxia-terlangiectasia-Rad3-related, DNA-dependent protein kinase, and poly-ADP-ribose polymerase could also be targeted with a siRNAs, which represent novel strategies for overcoming chemotherapy resistance (61–64).

#### c-MET

The immunosuppressive potency of the glioblastoma microenvironment may be a function of tumor invasiveness and epithelial-mesenchymal transition (EMT), in which c-MET plays a key role. c-MET was recently shown to mediate EMT *via* activation of Wnt/β-catenin signaling (65). MiR-128-3p targeting c-MET inhibited glioblastoma migration and invasion and enhanced TMZ therapeutic efficacy *in vivo* (66). Other miRNAs such as miR-34a, miR-144-3p, and miR-562 have been reported to exert activity against glioblastoma proliferation and invasion by also targeting c-MET (66–68). Hence, these therapeutic miRNAs and c-MET siRNAs could be used to treat glioblastoma by means of suppressing tumor invasion and EMT.

#### Chitinase-3-Like-1

GIM mediate immunosuppression is mediated by the chitinase-3-like-1 (CHI3L1)/Gal3-PI3K/ATK/mTOR axis. Chen et al. showed that inhibiting CHI3L1 complexes reversed GIM immunosuppression and delayed tumor progression (69). In other cancer models, genetic ablation of CHI3L1 *in vivo* reduced macrophage recruitment and increased effector T-cell infiltration in the tumor (70). Thus, siRNA targeting CHI3L1 is another potential therapy that could be beneficial to glioblastoma patients.

#### TGFβ

Overactivation of TGFβ signaling plays a critical role in reprogramming the glioblastoma to be immune suppressive and mediates immune escape and treatment resistance. In the glioblastoma microenvironment, a variety of dysfunctional cellular components and their interaction such as tumor, T, myeloid and NK cells are governed by the TGFβ signaling pathway. Consequently, the anti-TGFβ latency-associated peptide antibody can enhance antitumor immune responses in murine glioblastoma models (71); miR-142-3p targeting the TGFβR1 on M2 macrophages results in glioblastoma growth inhibition (28), and targeting the TGFβ-integrin axis improves NK cell antiglioblastoma activity (72). Anti-TGFβ RNA therapeutic represents a promising treatment avenue to be investigated in glioblastoma patients. A recent study showed that antisense oligonucleotides specifically targeting TGFβ_1_ and TGFβ_2_ exerts strong antiglioblastoma activity *in vitro* and *in vivo* (73).

#### S100A

S100A gene family members can modulate EMT, GSC stemness, and immune cell infiltration and are candidate therapeutic targets for glioblastoma patients (74). S100A4 is the most studied as a central player controlling EMT, stemness, and neutrophil infiltration. Its depletion downregulates the glioblastoma progression and treatment resistance (75, 76). Several microRNAs such as miR-124 (77), miR-149-3p (78), and miR-520c (79) have been identified as targeting S100A4, and their mimics resulted in antiglioblastoma activity *in vivo*. Therefore, these suppressor miRNAs and siRNAs targeting S100A4 could also be explored for therapeutic activity in glioblastoma patients.

## Targeting Strategies

### Antisense Oligonucleotides (ASOs)

Antisense oligonucleotides (ASOs) bind sequences specifically by Watson-Crick base pairing to the target RNA and regulate protein expression through RNase H mediated degradation and ribosome blockage by steric hindrance (80, 81). Significant advancement of oligonucleotide chemistry and numerous delivery platforms enhance ASO development and clinical application. Recently, the FDA has approved a few nucleic acid-based drugs, which stimulates greater interest in ASO therapeutic development. Currently, a variety of ASO drugs are being tested in clinical trials to treat cancer, infectious, and neurodegenerative diseases (82). Some ASO drugs target oncomiRs that promote tumorigenesis and metastasis. For example, RG-012, an anti-miR-21 ASO for Alport syndrome is being evaluated to ascertain if it decreases the rate of progression of renal fibrosis (83). Cobomarsen (MRG-106) is a miR-155 inhibitory ASO presently in phase II trials treating T cell leukemia and lymphoma (84). The anti-miR-21 and anti-miR-155 ASO strategy have been tested in preclinical models and showed potent anti-glioma efficacy (85, 86). Therefore, these anti–oncomiR ASOs can potentially be applied for the treatment of glioblastoma patients that have miR–21 and miR–155 dysregulation.

### Aptamers

Aptamers are short oligonucleotides that possess a 3–D distinct structure for their target recognition and binding. Systematic Evolution of Ligands by EXponential enrichment (SELEX) is the most used screening approach to identify specific aptamers binding a target with high affinity and selectivity. Their relatively small molecular weight (one–tenth that of monoclonal antibody) makes them more accessible to the glioblastoma microenvironment (87). In addition, aptamers are called chemical antibodies since they can be derived completely using chemical synthesis. Moreover, the low immunogenicity and long shelf life are advantageous features of aptamers for clinical application. Oligonucleotide aptamers are synthesized and assembled with cell–free automation enabling rapid and cost–effective production with minimal variation between batches. However, for clinical utility, they require further chemical modification for improving their *in vivo* half–life because of fast renal excretion and nuclease degradation. These modifications should have minimal effect on the affinity and specificity of the aptamers, and simultaneously improve their stability. Some promising modifications include inverted thymidine capping on the terminals, two hydroxyl group modifications in the ribose ring, the phosphodiester bond replacement and PEGylation (88). De La Fuente et al. identified several human and mouse specific RNA aptamers using tumor–associated myeloid cells as the targets *via* SELEX. These aptamers were specific to tumor–associated MDSCs in several cancer lineages including glioblastoma and had high binding affinity—highlighting their application as therapeutics targeting to the tumor microenvironment (89). Conjugating these MDSC–specific aptamers to a tumoricidal agent could significantly improve their potency.

### Conjugates of Aptamer and siRNA

Nucleotide aptamers and siRNAs share the same nucleic acid units, so base pair annealing, or covalent linkage can create an aptamer–siRNA chimera. These chimeras have great advantages over protein and cellular products: single component simplicity, small size, and easy manufacturing (90, 91). They also have less immunogenicity because the human immune system does not recognize nucleic acids as foreign molecules (92). Additionally, the siRNA portion in the chimeras can still be recognized and processed by Dicer with no compromised efficacy, resulting in their target mRNA degradation and protein depletion (93). The first aptamer–siRNA chimera designated PSMA aptamer–Plk1 siRNA was constructed in 2006. Since then, a number of aptamer–siRNA chimeras have been made with improved stability, targetting specificity and *in vivo* efficacy (94). A list of the examples for targeted RNAi potentially applicable to the glioblastoma and its associated microenvironment are presented in **Table 1**.

**Table 1:** Summary of aptamer–siRNA chimeras potentially applicable to the glioblastoma.

Formulation	Aptamer target	Target gene	Outcome	Reference
Aptamer–siRNA chimera	CTLA4	STAT3	Apoptosis of tumor cells and suppression of T–cell lymphoma outgrowth in immunodeficient mice	Hermann (95)
Aptamer–siRNA chimera	EpCAM	PLK1	Inhibition of EpCAM+ breast cancer growth in xenograft models	Gilboa–Geffen (96)
Aptamer dimer–siRNA chimera	4–1BB	mTOR complex 1 (mTORC1)	Inhibition of mTORC1 signaling in CD8+ effector T cells to induce a T–cell memory response and protective immunity by 4–1BB aptamer dimer activation	Berezhnoy (97)
Aptamer–siRNA chimera	αvβ3 integrin	Elongation factor 2	Inhibition of cell proliferation and the induction of apoptosis specifically in multiple cancer lineages including glioblastoma	Hussain (98)
Aptamer dimer–siRNA	4–1BB	CD25, Axin–1	Anti–tumor activity mediated by enhanced CD8+ T cell memory response in multiple syngeneic mouse models	Rajagopalan (99)
Aptamer–siRNA chimera	Nucleolin	SLUG/NRP1	Suppression of tumor cell invasion, growth, and angiogenesis	Lai (100)
Dox–aptamer–siRNA chimera	EpCAM	Survivin	Prolonged survival in mice bearing chemoresistant breast tumor	Wang (101) and Subramanian (102)
Aptamer–siRNA chimera	PDGFRβ	STAT3	Inhibition of glioma cell growth and angiogenesis *in vivo* in a xenograft mouse model	Esposito (103)
Aptamer–siRNA chimera	PDGFRα	STAT3	Inhibition of glioma cell viability	Yoon (104)
Aptamer–siRNA chimera	EpCAM	PKC_I_	Inhibition of PRKCI amplified ovarian cancer cell proliferation and xenograft model tumor growth	Rehmani (105)

### Aptamer–siRNA Therapeutic Overcoming Resistance to Immune Checkpoint Blockade

In spite of significant successes of immune checkpoint blockade (ICB) in treating cancer patients, thus far, this immunotherapy approach has minimal efficacy for the vast majority of glioblastoma patients secondary to a wide variety of mechanisms such as mutations in the antigen presentation pathway and the IFN–γ signaling pathway (106). Protein tyrosine phosphatase (PTPN2) has been identified by *in vivo* CRISPR screening as a new target–mediating resistance to ICB immunotherapy. Knockdown of PTPN2 results in enhanced ICB therapeutic efficacy by promoting antigen presentation and IFN–γ signaling in tumor cells (107). PTPN2 is overexpressed in glioblastoma and its expression associates with IDH wild–type expression and the mesenchymal subtype that indicates a worse prognosis. Furthermore, there is an inverse relationship between PTPN2 and an inflammatory response in glioblastoma (108). Thus, we believe that a PTPN2 siRNA–tumor–specific aptamer therapeutic may provide an important strategy to overcome ICB resistance of glioblastoma patients.

Another target mediating ICB resistance is A–to–I editing of interferon–inducible RNA species (ADAR1) that encodes an adenosine deaminase that inhibits the sensing of endogenous double–stranded RNAs (dsRNAs), and subsequently hinders antitumor immunity. Thus, ADAR1 inhibition can improve patient responses when combined with PD–1 blockade by overcoming the resistance mechanism of nonresponding to endogenous dsRNAs (109). ADAR1 is found highly active in glioma tissues and cells and essential for the maintenance of gliomagenesis (110), so we propose that ADAR1 can be a potential target inhibited by tumor cell specific aptamer–ADAR1 siRNA conjugates.

### Neoantigen Induction in Glioma Cells by Aptamer–siRNA

One of the major challenges in developing effective cancer immunotherapy is to identify tumor–specific and immunogenic neoantigens to stimulate a robust and durable immune response. There are several approaches to induce neoantigens in tumor cells *in situ* by aptamer–siRNAs specific to unique pathways that trigger the expression of neoantigens. The first pathway is nonsense–mediated messenger RNA decay (NMD), which is a highly conserved surveillance mechanism in mammal cells that prevents the translation of mRNAs with a premature stopping codon. NMD inhibition using tumor–specific oligonucleotide aptamer–targeted delivery of siRNAs to NMD–associated molecules such as SMG1 and UPF2 results in the expression of *de novo* antigens encoded by the premature stopping codon–containing mRNAs and their immune–mediated tumor rejection in metastatic and subcutaneous tumor models (111). This strategy is readily applicable to human glioblastoma as NMD pathway is important for gliomagenesis detection (112) and SMG1 mRNA expression is present in glioma cells (TCGA_GBM data). The second one is to target the transporter associated with antigen processing (TAP). Genetic ablation results in drastically enhanced presentation of new MHC class I–restricted epitopes independent of TAP. These induced new antigen epitopes form MHC–peptide complexes for engaging and activating CD8+ T cells capable of killing TAP–deficient tumor cells. Administration of TAP siRNAs conjugated to a tumor–targeting nucleolin aptamer (AS1411) has been shown to exert antitumor activity in multiple mouse tumor models (113). Both TAP and nucleolin are expressed in gliomas, making AS1411 and TAP siRNA conjugates an appealing candidate of the RNA–based immune therapy for treating glioblastoma patients with preclinical efforts underway by our group.

### Aptamer–siRNA Targeting STAT3 Signaling in the Tumor Microenvironment

STAT3 has been shown to be a signaling hub in tumor cells as well as tumor–associated immune cells (114, 115). In the glioblastoma microenvironment, STAT3 is persistently activated in glioma cells, myeloid, and T cells and promotes tumor cell survival, proliferation, invasion, and immunosuppression (26, 116, 117). Due to CTLA4 upregulation on tumor–infiltrating CD8+ T cells, a CTLA4‐targeting aptamer STAT3 siRNA chimera was created that triggers CD8+ T cell reactivation in the tumor microenvironment. Additionally, this chimera inhibits tumor–infiltrating Tregs and shows significant antitumor efficacy in multiple primary and metastatic tumor models (95). The Yu group has generated a DNA aptamer CpG1668–STAT3 siRNA chimera linked by a C3 carbon chain, which preserved the immunostimulatory properties of CpG1668 and at the same time does not interfere with Dicer processing of siRNA, thereby contributing to synergistic antitumor effects (118). Other aptamers have been tested including: (1) a STAT3 siRNA that successfully induces antitumor effects in glioblastoma when conjugated with a PDR3 aptamer against PDGFRα (104), and (2) a PDGFRβ–specific aptamer–siRNA chimera designated Gin4.T–STAT3 that could efficiently antagonize STAT3 in PDGFRβ+ GBM xenografts (103, 119).

### Aptamer–siRNAs Targeting Highly Enriched Chemokines and Cytokines in the Glioblastoma Microenvironment

Two major cell components in the glioblastoma microenvironment responsible for tumor escape from immune surveillance include GSCs and GIMs (26, 116, 120). Osteopontin (OPN), a key molecule–mediating immune suppression in this setting, is highly expressed in both GSCs and GIMs. It is a secreted phosphoprotein chemokine that also operates intracellularly with both forms playing important roles in tumor growth and metastasis (121). Our data indicate that highly expressed OPN in the glioblastoma microenvironment is indispensable for macrophage infiltration. We have further shown that both tumor–derived OPN and nontumor–derived OPN are essential for glioblastoma development. A deficiency of OPN in either glioblastoma cells or immune cells results in a marked reduction in numbers of immune suppressive M2 macrophages and enhanced T–cell effector function (87). As such, OPN is an attractive therapeutic target specific to the glioblastoma microenvironment. Interestingly, periostin sharing the same RGD functional motif with OPN is secreted from GSCs and correlates with GIM infiltration in human glioblastoma. Periostin depletion diminishes the tumor supportive M2 type of GIMs in xenografts (122). CCL2, another chemokine highly enriched in the glioma microenvironment, is important for attracting both CCR2+Ly–6C+ monocytic MDSCs and CCR4+ Tregs. CD163+ GIMs are a major source producing CCL2 in the glioblastoma microenvironment (123). GIM–derived CCL8 contributes to the stemness maintenance and invasion of glioblastoma cells through ERK1/2 pathway and its blockade significantly decreases invasion of glioma cells (124). Boeck et al. have also shown that IL–33 is another important chemokine–mediating GIM infiltration since its expression correlates with GIM density in human and mouse glioma tissues. Furthermore, both intracellular and secreted isoforms of IL–33 upregulate other chemokines that collectively recruit and transform peripheral innate immune cells to create an immunosuppressive environment (125). Bispecific aptamer siRNA conjugates to the aforementioned chemokines are plausible strategies and could be developed.

### Bispecific Aptamers to Elicit Antitumor Immunity

Bispecific aptamers, composed of two aptamers, exhibit concurrent binding to two different entities such as antigens. Absence of costimulatory signal in the tumor microenvironment results T–cell energy (126). Accordingly, an aptamer specific to 4–1BB receptor has been developed to target and activate tumor–infiltrating T cells (127). Pastor et al. generated the first bispecific aptamers consisting of a bivalent 4–1BB aptamer and a tumor–specific PSMA aptamer, enhancing the conjugate delivery to the tumor niche and activation of costimulatory responses. Profound antitumor activity of a 4–1BB–PSMA aptamer chimera has been observed in multiple immune competent mouse models including colon cancer and melanoma lung metastasis when administered systemically (128). A similar strategy could be considered for glioblastoma but would require the selection of a subset of patients that express a given tumor antigen. Other bispecific aptamers have been engineered to specifically target CD28–expressing T cells in multidrug resistance–associated protein 1 melanoma that triggered prolonged survival of tumor–bearing mice. The associated immune mechanism included reactivation of tumor–infiltrating T cells *via* CD28 costimulation by CD28 aptamer binding and crosslinking (95). Schrand et al. synthesized another bivalent aptamer by fusing an agonistic 4–1BB aptamer with an aptamer specific to VEGF and showed effective targeting of the stroma in the tumor microenvironment. This 4–1BB–VEGF aptamer was capable of inducing the activation and expansion of CD8+ effector T cells and promoting T memory cell differentiation that prevented tumor recurrence across cancer lineages (129). Since VEGF expression is a common feature of glioblastoma, a strategy in which T cells are activated and expanded may induce glioma cell–specific killing (130), but this will require preclinical testing. A list of the examples for bispecific aptamers potentially applicable to the glioblastoma and its associated microenvironment is presented in **Table 2**.

**Table 2:** Summary of bispecific aptamers applicable to the glioblastoma treatment.

Formulation	Targeting aptamer	Effector aptamer	Outcome	Reference
Aptamer–aptamer	MRP1	CD28	Inhibition of tumor growth of melanoma–bearing mice	Soldevilla (126)
Aptamer dimer–aptamer	4–1BB	VEGF	Induction of potent antitumor immunity against multiple tumor types including glioma.	Schrand (129)
Aptamer–aptamer	EpCAM	CD44	Suppression of intraperitoneal ovarian cancer outgrowth much more significantly than single aptamers	Zheng (131)
Aptamer–aptamer	CD3	Liver cancer specific TLS11a	Effective inhibition of liver tumor growth and survival extension *via* binding hepatoma cells and T cells	Hu (132)
Aptamer dimer–aptamer	4–1BB	osteopontin	Increased median survival of glioma bearing mice with enhanced effector T cell infiltration	Wei (87)
Aptamer–siRNA chimera	Nucleolin	SLUG/NRP1	Synergistic inhibition of lung cancer cell invasion, tumor growth, and angiogenesis	Lai (100)
Aptamer dimer–aptamer dimer	CD16	Mucin 1	Recruitment of CD16+ immune cells to the MUC1+ tumor cells and enhancement of the immune cytotoxicity	Li (133) and Boltz (134)
Aptamer–aptamer	MRP1	ICOS	Strong antitumor immunity in combination with CTLA–4 blockade	Soldevilla (135)
Aptamer–aptamer	CD62L	PTK7	Linkage of T cells and tumor cells that induces tumor specific killing	Yang (130)
Aptamer–aptamer–gold nanocarrier	Nucleolin	c–MET	Enhanced anti–gastric and lung cancer efficacy	Lee (136)

### RNA Nanocarrier Delivery Systems

Aptamer–siRNA chimeras and bispecific aptamers are one of the most efficient strategies for target delivery modules because of their high specificity and binding affinity, fully automated synthesis, and great potential for clinical application. Nonetheless, a major obstacle needs to be overcome for delivery of siRNAs into the cytoplasm of the targeted tumor and/or immune cells. One hurdle for the efficient delivery of aptamer–siRNA chimeras to the cytoplasm is the negative charge of nucleotides and endosomal degradation. A strategy that could overcome this limitation is to embed the cell–specific aptamers into siRNA encapsulating nanoparticles, which improve the delivery efficacy of naked siRNAs passing through the cellular barriers.

### Natural Nanocarriers

Many cell types in the glioblastoma microenvironment interact with each other through microvesicles and exosomes (137). As a natural system of miRNA delivery, these vesicles can be secreted from genetically engineered miRNA overexpressing cells or generated from exosomes transfected with miRNAs (138, 139). Glioblastoma cells or stem cells, for example, could be genetically modified to express exogenous tumor suppressor miRNAs and the elaborated exosomes with the tumor–suppressor miRNA used as a therapeutic product. Proof–of–principal preclinical studies using this type of strategy have been shown to reduce the tumor burden and have potential clinical utility (140). Although there are other contents in the exosomes such as other RNA and protein molecules, this composition can be altered by the cell status and signaling stimuli (141). As such, these natural vesicles may be an excellent RNAi carrier system (142). Continuing efforts are warranted to improve capacity of these exosome nanocarriers for passing the BBB and efficient delivery of their siRNA cargo to the glioblastoma microenvironment.

Other types of natural modifications include conjugation to chitosan, a natural polysaccharide composed of repeating *N*–acetyl–glucosamine and glucosamine units. The cationic charge of chitosan can rapidly form complexes with negatively charged nucleic acids. Furthermore, highly reactive amino and hydroxyl groups of chitosan allow for chemical modification and linkage of cognate ligands (143). Noh et al. demonstrated that systemic administration of EGFL6 siRNA—chitosan nanoparticles were delivered to endothelial ovarian cancer cells and markedly inhibited tumor progression (144).

For effective glioblastoma therapy, nanoparticles that are delivered systemically must have tumor and/or immune specificity with little measurable side effects. More recently, RNA nanocarriers have gained attention as a versatile natural platform of nanoassembly and construction. The three–strand packaging RNA complex in the polyhedra bacteriophage phi29 is self–assembled and highly dynamic. This unique feature is utilized to generate a variety of RNA nanoparticles with a wide range of specific sizes and shapes. The pRNA–3WJ motif is a three–RNA–strand scaffold that is capable of targeting intracranial gliomas in mice (145).

### Synthetic Nanocarriers

Lipid–formulated nanoparticles have intensively been utilized in laboratory studies and clinical trials for RNA therapeutics delivery because of the ease of manufacturing and high biocompatibility (146). Sun et al. developed a novel liposome system simultaneously delivering survivin siRNA and paclitaxel to the glioblastoma. Specifically, a CD133–specific RNA aptamer and a low–density lipoprotein receptor–related protein were integrated into the exterior membrane of the liposomes, resulting in dual targeting ability to bind glioblastoma cells and endothelial cells in the tumor microenvironment. This lipid nanoformulation could enrich in the tumor niche *via* efficiently binding to the low–density lipoprotein receptor expressing endothelial cells, and selectively induce apoptosis of CD133+ GSCs as well as endothelial stroma cells (147). Another similar liposome siRNA delivery carrier has been developed to treat breast cancer by means of CD44 aptamer targeting. These liposomes were found to efficiently inhibit CD44+ tumor outgrowth *in vivo* (148). CD44 is also found overexpressed in glioma cells and a common GSC surface marker (149). As such, an anti–CD44 aptamer–equipped siRNA liposome delivery system may be applicable to glioblastoma.

A critical determinant of successful delivery of RNA is the prevention of nuclease degradation. Polymeric nanocarriers have been extensively utilized for the protection of miRNAs and siRNAs due to their positive charge (150). Recently, cationic polymers were broadly used to form stable complexes with negatively charged RNA. Among them, polyethyleneimine is the most commonly used for nucleic acid delivery, but its clinical application is hampered by its inherent toxicity. One alternative approach is to use hybrid polymers such as PEI–chitosan hybrid nanocarriers that show an improved safety profile (151). Another strategy is to link polyethyleneimine polyplexes with brain–targeting rabies virus glycoprotein. These nanoparticles have shown both effective brain tumor targeting and low toxicity (152).

## Conclusion and Future Perspective

Despite enormous and continuous efforts for developing new and combinational treatment strategies for glioblastoma, there has been minimal improvement in survival. The brain tumor microenvironment is a key driver that promotes and regulates tumor initiation and progress and mediates the treatment resistance in both primary and metastatic brain malignancies. Molecular dissection into the protumorigenic functions of single elements of the brain tumor microenvironment has resulted in the discovery of a number of promising noncoding RNA therapeutic targets. These RNA therapeutics include miRNAs, siRNAs, and aptamers and are emerging as a novel avenue to treat brain cancer patients. Since miRNAs can act upon multiple targets and pathways regulating immune suppression and chemoresistance, they may be more effective in treating the malignancies such as glioblastoma that are highly heterogeneous. Nonetheless, one major challenge remains for clinical application for treating glioblastoma is the ability of an agent to cross the BBB. This issue is alleviated by their small molecular weight and compact size, which can be further enhanced with nanocarrier–specific targeted delivery. Increasingly sophisticated nanoparticle systems, also relying on targeting moieties for BBB penetration and/or improved target cell transfection efficacy, may provide an avenue toward clinical application. On the other hand, it should be noted that too complicated systems based on multiple components may prohibit drug approval and transition into the clinic.

The unique characteristics of aptamers make them highly attractive for targeted therapy of glioblastoma and the other malignancies. A given aptamer can be conjugated to another aptamer, siRNA or miRNA, which leads to the production of multimodal chimeric therapeutics with novel functions enabling simultaneous targeting of numerous molecules and cell subsets in the glioblastoma microenvironment. Their small size and simple structure may have key advantages superior for passing through the BBB and gaining access to the brain tumor when administered systemically.

## Author Contributions

JW provided ideas for the project and wrote the initial draft. ABH, GAC and EG completed the final version. All authors contributed to the article and approved the submitted version.

## Funding

Funding was provided by the Ben and Catherine Ivy Foundation, the MD Anderson Cancer Center Provost Fund and NIH grant CA120813.

## Conflict of Interest

GC is one of the scientific founders of Ithax Pharmaceuticals.

The remaining authors declare that the research was conducted in the absence of any commercial or financial relationships that could be construed as a potential conflict of interest.

## Publisher’s Note

All claims expressed in this article are solely those of the authors and do not necessarily represent those of their affiliated organizations, or those of the publisher, the editors and the reviewers. Any product that may be evaluated in this article, or claim that may be made by its manufacturer, is not guaranteed or endorsed by the publisher.
